# Down-regulation of lncRNA DNAJC3-AS1 inhibits colon cancer via regulating miR-214-3p/LIVIN axis

**DOI:** 10.1080/21655979.2020.1757224

**Published:** 2020-04-30

**Authors:** Bing Han, Yang Ge, Junpeng Cui, Baolin Liu

**Affiliations:** Department of General Surgery, Shengjing Hospital of China Medical University, Shenyang, Liaoning, People’s Republic of China

**Keywords:** Colon cancer, DNAJC3-AS1, miR-214-3p, LIVIN, NF-κB

## Abstract

Long non-coding RNAs (lncRNAs) play a key role in the development and metastasis of cancer. However, the biological role and clinical significance of lncRNA DNAJC3-AS1 in the development of colon cancer is still unknown. In this study, the effects of DNAJC3-AS1 on cell proliferation, migration, and invasion were evaluated by MTT assay, wound-healing assay, and transwell assay, respectively. The relationship between DNAJC3-AS1, miR-214-3p and LIVIN was predicted by the online software and confirmed by dual-luciferase reporter assay. We found that the down-regulation of DNAJC3-AS1 inhibited the proliferation of colon cancer cells and induced growth arrest. Down-regulation of DNAJC3-AS1 also inhibited the migration, invasion, and epithelial-mesenchymal transition (EMT) of colon cancer cells. Moreover, miR-214-3p can bind to DNAJC3-AS1, and knockdown of DNAJC3-AS1 increased miR-214-3p expression in colon cancer cells. LIVIN was identified as a target of miR-214-3p. The up-regulation of miR-214-3p inhibited the protein expression of LIVIN and suppressed the activation of the NF-κB signaling pathway. Besides, down-regulation of DNAJC3-AS1 reduced cell viability, invasion, and EMT of colon cancer cells, while miR-214-3p inhibitor could reverse these effects. The expression of LIVIN and the activation of the NF-κB signaling pathway were suppressed by down-regulating DNAJC3-AS1, while these effects could be restored by miR-214-3p inhibitor. These findings suggested that DNAJC3-AS1 may promote colon cancer progression by regulating the miR-214-3p/LIVIN axis. DNAJC3-AS1 may serve as a new biomarker and therapeutic target for colon cancer, stimulating new research directions and treatment options.

## Graphical Abstract

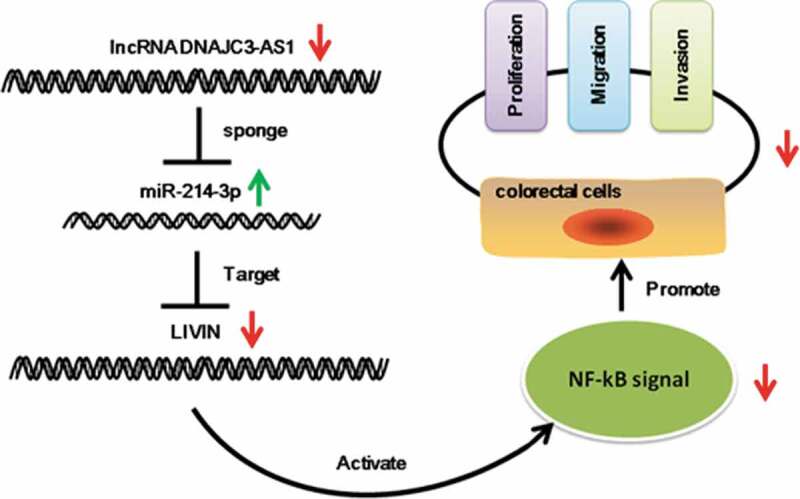

## Introduction

Colon cancer is one of the malignant cancers with the highest morbidity and mortality in the world, and it seriously threatens people’s health and life [1]. The incidence of colon cancer varies worldwide. The rate of colon cancer is higher in developed countries than in non-industrialized countries, and in men than in women [2]. Besides, the incidence of colon cancer continues to rise in adults under the age of 50 and increases with age [2]. However, the advances in cancer diagnostics and treatments have not significantly extended the overall survival of patients with colon cancer [3]. The main reason is that current treatments lack effective biomarkers [4].

LncRNAs are non-coding RNAs with a transcript longer than 200 nucleotides and have more cell-type specificity compared with mRNAs [5]. Previous studies have demonstrated that lncRNAs participate in the regulation of various biological processes in cells [6–8]. MicroRNA (miRNA) is a class of endogenous single-stranded non-coding RNA molecules with a length of about 22 bp [9]. miRNAs can also participate in various biological processes such as cell proliferation, differentiation, apoptosis, and angiogenesis through regulating downstream target genes [10–12]. miRNAs are closely related to the occurrence and development of many cancers, including colon cancer [13]. The hypothesis of competitive endogenous RNA (ceRNA) was proposed in 2011 [14], suggesting a new regulatory mechanism of miRNAs and lncRNAs. The ceRNA hypothesis indicates that some endogenous RNAs contain certain miRNA binding sites, and they can regulate the expression of miRNAs target mRNAs through regulating miRNA expression [14].

LncRNAs are considered to play a vital role in colon cancer development. For example, lncRNA MALAT1 can mediate HMGB1 by sponging miR-129-5p to promote colon cancer [15]. LncRNA HOTAIR may promote colon cancer by sponging miR-34a [16]. LncRNA HULC mediates the expression of RTKN through miR-613, thereby playing a tumorigenic role in colon cancer [17].

Recent research indicates that lncRNA DNAJC3-AS1 can promote the progression of osteosarcoma [18]. However, its role in colon cancer has not been reported. Bioinformatic prediction indicated that DNAJC3-AS1 contains miR-214-3p binding sites. Many studies show that miR-214-3p could inhibit colon cancer [19–21]. Moreover, we predicted that miR-214-3p could bind to LIVIN. Researchers found that over-expressing LIVIN can activate the NF-κB signaling pathway and promote the proliferation, migration, invasion and epithelial-mesenchymal transition (EMT) of colon cancer cells [22]. In contrast, inhibiting the expression of LIVIN can inhibit the malignant phenotype of colorectal cells [22].

The aim of the current study is to investigate the role of DNAJC3-AS1 on the progression of colorectal cancer and reveal the possible mechanisms. Our results demonstrate that the down-regulation of DNAJC3-AS1 suppressed the proliferation, migration, and invasion of colorectal cells. Further, DNAJC3-AS1 functions as a sponge to regulate the miR-241-3p level, thus, affect the malignant activity of colorectal cells via the miR-214-3p/LIVIN axis.

## Materials and methods

### Cell culture and transfection

Colon cancer cells (SW620, SW480, Caco-2, and LoVo) and 293 T were purchased from Procell (Wuhan, China). HT29 was purchased from Zhongqiao xinzhou Biological Technology Co., Ltd (Shanghai, China). Caco-2 was cultured in Minimum Essential Medium (Gibco, USA) supplemented with 10% fetal bovine serum (Hyclone, USA). LoVo was cultured in Ham’s F12 K Medium (Procell, China) containing 10% fetal bovine serum. SW620 and SW480 were cultured in Roswell Park Memorial Institute 1640 Medium (Gibco, USA) supplemented with 10% fetal bovine serum. HT29 and 293 T were cultured in Dulbecco’s Modified Eagle’s medium (Gibco, USA) supplemented with 10% fetal bovine serum. All cells were cultured in an atmosphere at 37 °C with 5% CO_2_.

DNAJC3-AS1 siRNA (si-DNAJC3-AS1), and negative control siRNA (si-NC) were synthesized by JTS scientific (Wuhan, China). miR-214-3p mimics, miR-214-3p inhibitors and negative controls were obtained from JTS scientific (Wuhan, China). Colon cancer cells were transfected using the Lipofectamine 2000 transfection reagent (Invitrogen, USA) according to the manufacturer’s protocol.

### The online analysis of DNAJC3-AS1

A web tool GEPIA(http://gepia.cancer-pku.cn/detail.php)^23^ was used to analyze the DNAJC3-AS1 expression and overall survival in colorectal cancer patients. The expression analysis was performed as follows: Gene: DNAJC3-AS1, |Log2 FC| Cutoff:1, p-value Cutoff:0.05, Datasets Selection: COAD, log2(TPM + 1) for log-scale: Yes, Matched Normal data: Match TCGA normal and GTEx data. The correlation of overall survival analysis was performed as follows: Gene: DNAJC3-AS1, Methods: Overall Survival, Group Cutoff: Median, Hazards Ratio (HR): Yes, 95% Confidence Interval: Yes, Datasets Selection: COAD.

### RNA extraction and real-time PCR (qPCR)

Total RNA was extracted from colon cancer cells using RNA simple Total RNA Kit (TIANGEN, China). Then cDNA was synthesized by Moloney Murine Leukemia Virus reverse transcriptase (M-MLV, TIANGEN, China). The real-time PCR (qPCR) was carried out with 2 × Taq PCR MasterMix (TIANGEN, China). The primers for qPCR were shown in Table 1. qPCR data were collected and analyzed using Exicycler 96 software (Bioneer, Korea).

**Table 1.** Primer sequences used for qPCR.

	Forward primer	Reverse primer
DNAJC3-AS1	TTGCTCTAAGGCAGGTAGGTAA	GGGTCTTCCACAATCGCTCT
LIVIN	GAGGTGCTTCTTCTGCTATGGG	GCTGCGTCTTCCGGTTCTT
GAPDH	GACCTGACCTGCCGTCTAG	AGGAGTGGGTGTCGCTGT
miR-214-3p	AGCAGGCACAGACAGGCA	GCAGGGTCCGAGGTATTC
U6	GCTTCGGCAGCACATATACT	GTGCAGGGTCCGAGGTATTC

### MTT assay

Cell proliferation of colon cancer cells was detected by MTT assay according to the manufacturer’s instruction. Cells were cultured in the 96-well plate and were detected at 24, 48, 72 and 96 hours after transfection. MTT (KeyGEN BioTECH, China) was put into the medium and incubated at 37°C for 5 hours. The absorbance (OD) was measured using EXL-800 Microplate Reader (BIOTEK, USA) at 570 nm.

### 5-ethynyl-2ʹ-deoxyuridine (EdU)

Cell proliferation was assayed using the Click-iT EdU Imaging Kit (KeyGEN BioTECH, China) according to the manufacturer’s instructions, and then stained with Hoechst 33,342 (Invitrogen, USA) for 15 min. These results were from three independent experiments.

### Flow-cytometry analysis

For cell cycle analysis, we used the Cell Cycle Kit (Beyotime, China). After transfection, cells were was harvested and fixed with 70% ethanol at 4°C for 2 h. Then, cells were centrifuged at 1000 g for 5 min to collect these cells. Next, these cells were incubated with 25 μl of propidium iodide (PI) liquid and 10 μl RNase A at 37°C for 30 min. NovoCyte Flow Cytometer (ACEA Biosciences, China) was used to measure the cell cycle.

### Wound-healing assay

After transfection, the monolayer cell was scratched with a 200 μl pipette tip, and the medium was replaced with a serum-free medium. Each scratch wound was recorded with a microscope at the same position at 0 h and 24 h, respectively. The experiments were conducted in triplicate.

### Transwell assay

The capacity of cell invasion was assessed by transwell assay. First, the transwell chamber was covered with 40 μl Matrigel (BD, USA). The colon cancer cells were seeded on the upper chamber and incubated with serum-free culture medium. Next, 800 μl medium was added in the lower chamber with 30% serum. After a 48-h culture, the colon cancer cells in the upper chamber were washed twice. The lower cells were fixed with 4% paraformaldehyde (Aladdin, China) for 25 min and stained with 0.4% crystal violet stain (Amresco, USA) for 5 min. Five randomly microscopic fields were selected for analysis.

### Immunofluorescence (IF)

Cells were seeded on the coverslips and cultured for proper cell density. The cells were blocked with FBS after fixing by 4% paraformaldehyde (Aladdin, China). Then, the cells were incubated with the E-cadherin rabbit antibody (proteintech, China) overnight at room temperature. Next, these samples were washed with PBS and incubated with fluorescein Cy3-conjugated secondary antibodies (Beyotime, China). Then, the samples were counterstained with DAPI (Aladdin, China). Images were captured using the fluorescence microscope (Olympus, Japan).

### Dual-luciferase reporter assay

DNAJC3-AS1 wild-type luciferase plasmid containing potential miR-214-3p binding sites (wt-DNAJC3-AS1) and mutated versions of these sites (mut-DNAJC3-AS1) were obtained from GenScript (Nanjing, China). miR-214-3p/NC mimic (JTS scientific, China) was co-transfected with wt/mut DNAJC3-AS1 into 293 T cells using Lipofectamine 2000 reagent (Invitrogen, USA). Moreover, LIVIN wild-type luciferase plasmid containing potential miR-214-3p binding sites (wt-LIVIN) and mutated versions of these sites (mut-LIVIN) were obtained from GenScript (Nanjing, China). After 48-h transfection, renilla and firefly luciferase activities were tested by the Dual-Luciferase Reporter assay system (Promega, USA).

### Western blot analysis

Cells were lysed in RIPA buffer (Solarbio, China). BCA protein assay kit (Solarbio, China) was used to assay the protein concentrations. Sodium dodecyl sulfate-polyacrylamide gel electrophoresis (Solarbio, China) was used to separate proteins. Then, these proteins were transferred onto polyvinylidene fluoride membrane (Millipore, USA). After blocking with 5% BSA (Biosharp, China) in PBST (PBS with 0.05% Tween-20), membranes were incubated with E-cadherin rabbit antibody (Proteintech, China), N-cadherin rabbit antibody (Proteintech, China), LIVIN rabbit antibody (Proteintech, China), p-IκB rabbit antibody (Affinity, China), IκB rabbit antibody (Affinity, China), p-P65 rabbit antibody (Affinity, China), P65 rabbit antibody (Affinity, China) and GAPDH mouse antibody (Proteintech, China) overnight at 4°C, respectively. Then, these membranes were incubated with goat anti-rabbit IgG secondary antibody (Solarbio, China) and goat anti-mouse IgG secondary antibody (Solarbio, China) at 37°C for 1 h. The antigen-antibody reaction was observed by the ECL assay (Solarbio, China).

### Statistical analysis

GraphPad Prism 8 was used to analyze experimental data. The differences between 2 groups were analyzed by the T-test. Multiple groups were compared by one-way ANOVA. Data were presented as mean ± standard deviation (SD) from at least three independent experiments. The P-value <0.05 was considered statistically significant.

## Results

### DNAJC3-AS1 may involve in the development of colon cancer

**Figure 1.**
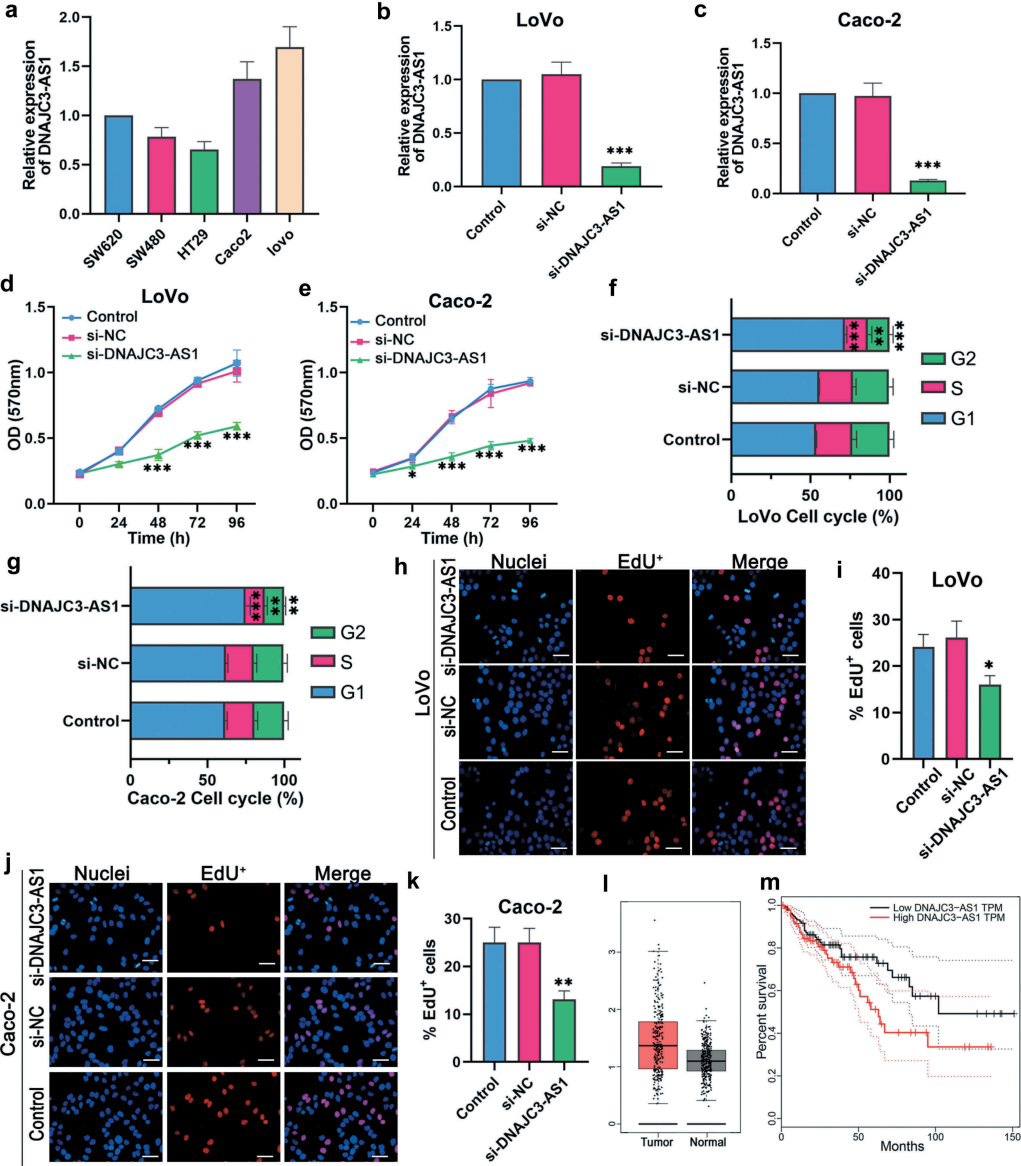
DNAJC3-AS1 may involve in the development of colon cancer. (a) The expression of DNAJC3-AS1 in colon cancer cell lines was determined by qPCR. (b and c) The expression of DNAJC3-AS1 was evaluated after the down-regulation of DNAJC3-AS1 in LoVo cells (b) and Caco-2 cells (c). (d and e) The MTT assay was performed to determine the effect of DNAJC3-AS1 on cell proliferation of LoVo cells (d) and Caco-2 cells (e). (f and g) The flow-cytometry assay was performed to confirm the effect of DNAJC3-AS1 on cycle distribution in LoVo cells (f) and Caco-2 cells (g). (h-k) Immunofluorescence staining was performed to detect the effect of DNAJC3-AS1 on the EdU^+^ cells in LoVo cells (h, i) and Caco-2 cells (j, k). Scale bar = 50 μm. (l) The differential expression of DNAJC3-AS1 mRNA between colon cancer tissues (n = 275) and normal samples (n = 349) from GEPIA analysis. The red box is colon cancer tissues, and the gray box is the normal tissues. (m) Elevated expression of DNAJC3-AS1 indicated poor clinical outcome for colon cancer patients. The overall survival curves of colon cancer patients with high or low DNAJC3-AS1 expression were plotted from the GEPIA database. Data is shown as mean ± SD, ** p < 0.01, *** p < 0.001.

We measured the expression of DNAJC3-AS1 in five colon cancer cells (SW620, SW480, HT29, Caco-2, and LoVo). The highest level of DNAJC3-AS1 was found in LoVo cells, followed by Caco-2 cells (Figure 1(a)). Further experiments showed that the level of DNAJC3-AS1 was significantly down-regulated in LoVo cells after transfection with si-DNAJC3-AS1 (Figure 1(b)). Consistently, the level of DNAJC3-AS1 was significantly down-regulated in Caco-2 cells after transfection with si-DNAJC3-AS1 (Figure 1(c)). Next, the MTT assay indicated that the down-regulation of DNAJC3-AS1 could inhibit the proliferation of LoVo cells (Figure 1(d)) and Caco-2 cells (Figure 1(e)). Flow-cytometry results showed that the percentage of cells in the G1 phase was markedly increased in LoVo cells (Figure 1(f)) and Caco-2 cells (Figure 1(g)) after transfection with si-DNAJC3-AS1. Down-regulation of DNAJC3-AS1 could decrease the S and G2 phases in LoVo cells (Figure 1(f)) and Caco-2 cells (Figure 1(g)). Additionally, the percentage of EdU^+^ cells was significantly decreased in LoVo cells (Figure 1(h,i)) and Caco-2 cells (Figure 1(j,k)) by down-regulating DNAJC3-AS1. The Gene Expression Profiling Interactive Analysis (GEPIA, http://gepia.cancer-pku.cn/) was used to analyze the differences of DNAJC3-AS1 in colon cancer patients and the effect of DNAJC3-AS1 on overall survival. The expression of DNAJC3-AS1 was markedly up-regulated in colon tumor tissues compared to normal colon tissues (Figure 1(l)). Kalpen-Meier survival plot showed that the patients with high DNAJC3-AS1 had shorter overall survival than those with low DNAJC3-AS1 (Figure 1(m)). Together, our results suggested that the down-regulation of DNAJC3-AS1 could inhibit the proliferation of colon cancer cells, and DNAJC3-AS1 may play a vital role in colon cancer development.

### DNAJC3-AS1 affected the cell migration, invasion, and EMT of colon cancer cells

**Figure 2.**
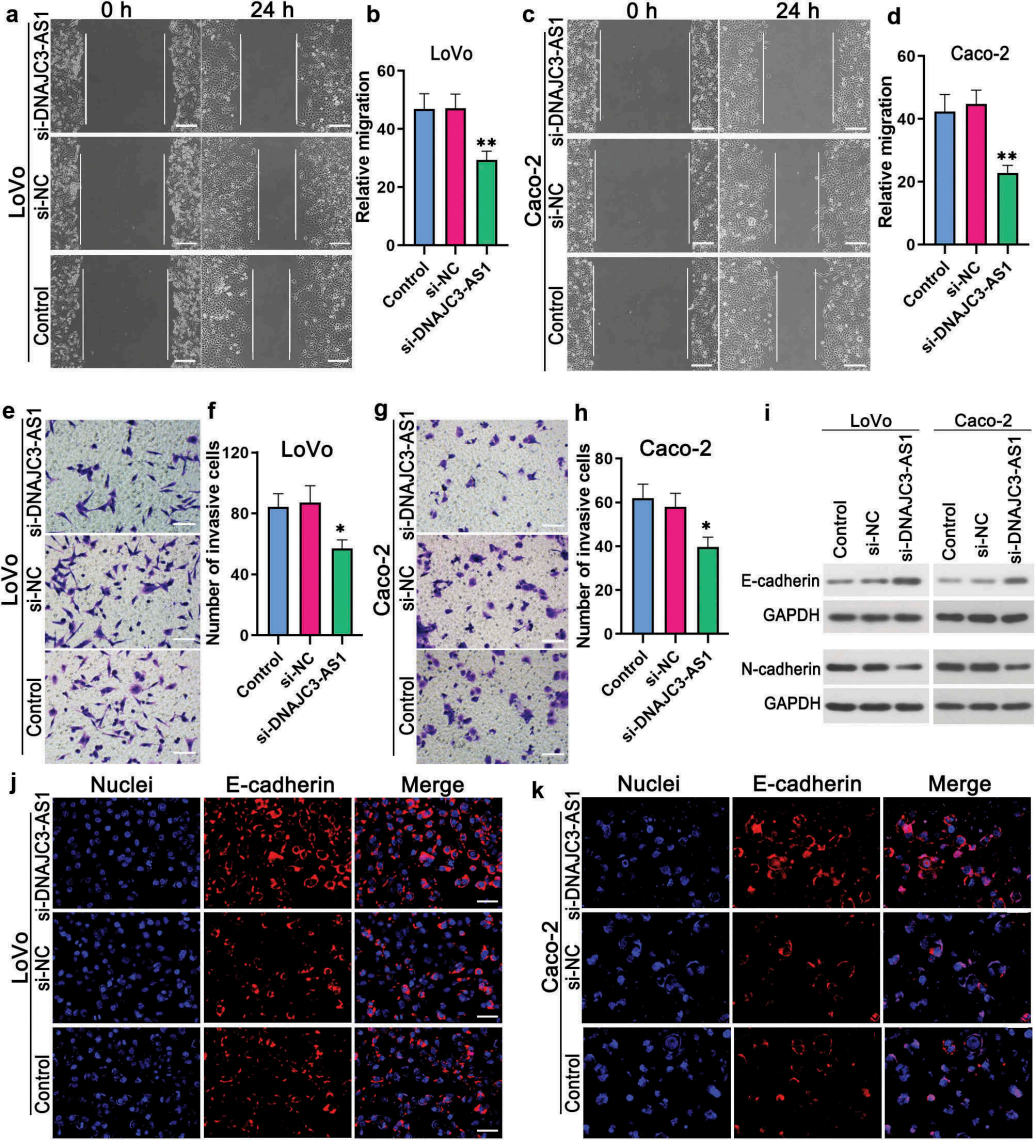
DNAJC3-AS1 affected the cell migration, invasion, and EMT of colon cancer cells. (a-d) Wound-healing assay was performed to evaluate the effect of DNAJC3-AS1 on the migration of LoVo cells (a, b) and Caco-2 cells (c, d). Scale bar = 200 μm. (e-h) Transwell assay was performed to detect the effect of DNAJC3-AS1 on the invasion of LoVo cells (e, f) and Caco-2 cells (g, h). Scale bar = 100 μm. (i) Western blot was performed to detect the protein expression of E-cadherin and N-cadherin in LoVo cells and Caco-2 cells. (j and k) Immunofluorescence staining was performed to examine the expression of E-cadherin in LoVo cells (j) and Caco-2 cells (k). Scale bar = 50 μm. Data is shown as mean ± SD, * p < 0.05, ** p < 0.01.

Tumor metastasis is one of the most important reasons for the failure of colon cancer treatment [24]. We performed the wound-healing assay to detect the migration ability of colon cancer cells. The migration rate of LoVo cells transfected with si-DNAJC3-AS1 was lower than that of LoVo cells transfected with si-NC (Figure 2(a,b)). Down-regulation of DNAJC3-AS1 could decrease the migrate rate of Caco-2 cells (Figure 2(c,d)). Transwell assay results indicated that the down-regulation of DNAJC3-AS1 could decrease the invasion cell number of LoVo cells (Figure 2(e,f)) and Caco-2 cells (Figure 2(h,i)), suggesting that colon cancer cells with si-DNAJC3-AS1 had weaker invasion ability compared with colon cancer cells with si-NC. Western blot results showed that the expression of E-cadherin was enhanced by down-regulating DNAJC3-AS1 in LoVo cells and Caco-2 cells (Figure 2(i)). The levels of N-cadherin were decreased by down-regulating DNAJC3-AS1 in LoVo cells and Caco-2 cells (Figure 2(i)). Besides, immunofluorescence was used to determine the levels of E-cadherin in colon cancer cells. The knockdown of DNAJC3-AS1 could increase the expression level of E-cadherin in LoVo cells (Figure 2(j)) and Caco-2 cells (Figure 2(k)). These data revealed that the down-regulation of DNAJC3-AS1 could inhibit the migration, invasion, and EMT of colon cancer cells.

### DNAJC3-AS1 affected miR-214-3p and its related signaling pathway

**Figure 3.**
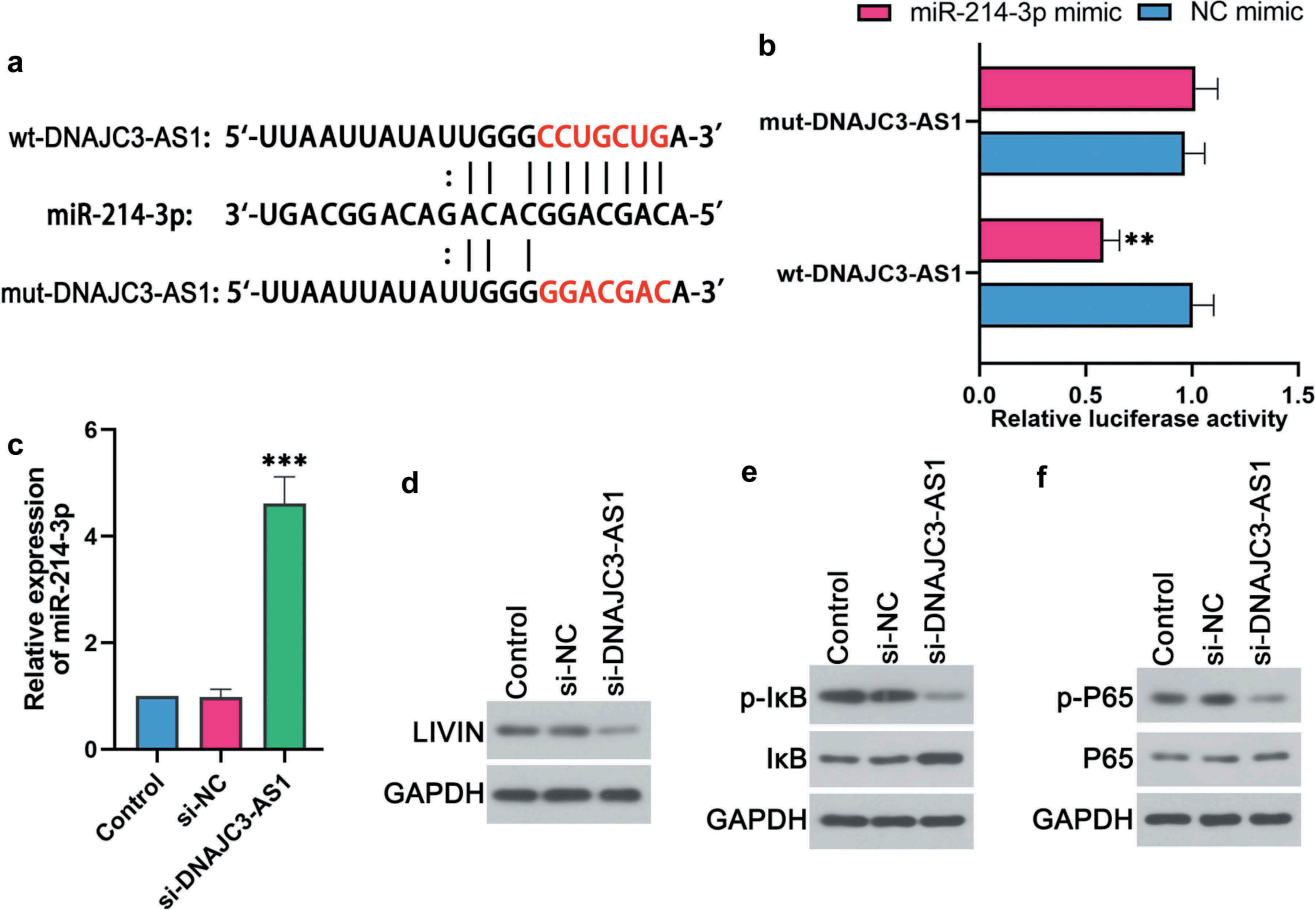
DNAJC3-AS1 affected miR-214-3p and its related signaling pathway. (a) Putative miR-214-3p binding and mutated sites in DNAJC3-AS1. (b) Dual-luciferase reporter assay was performed to determine the luciferase activity of 293 T cells. (c) The expression of miR-214-3p was evaluated after the down-regulation of DNAJC3-AS1 in LoVo cells. (d-f) The protein levels of LIVIN (d), p-IκB (e), IκB (e), p-P65 (f) and P65 (f) in LoVo cells were detected by western blot. Data is shown as mean ± SD, ** p < 0.01, *** p < 0.001.

Bioinformatic prediction (http://starbase.sysu.edu.cn) was shown in Figure 3(a). The prediction results indicated that DNAJC3-AS1 may bind to miR-214-3p (Figure 3(a)). Dual-luciferase reporter assay results showed that luciferase activity was significantly decreased in 293 T cells co-transfected with miR-214-3p mimic and wt-DNAJC3-AS1, while no significant difference was detected in 293 T cells co-transfected with miR-214-3p mimic and mut-DNAJC3-AS1 (Figure 3(b)). Then we used qPCR to measure the level of miR-214-3p. The knockdown of DNAJC3-AS1 enhanced the level of miR-214-3p in LoVo cells (Figure 3(c)). Western blot results indicated that the expression of LIVIN was significantly decreased by si-DNAJC3-AS1 (Figure 3(d)). Moreover, si-DNAJC3-AS1 inhibited the protein expression of p-IκB (Figure 3(e)) and p-P65 (Figure 3(f)) in LoVo cells. These indicated that miR-214-3p could bind to DNAJC3-AS1. DNAJC3-AS1 modulated the expression of miR-214-3p and LIVIN, and regulated the activation of NF-κBp65.

### miR-214-3p affected LIVIN and its related signaling pathway

**Figure 4.**
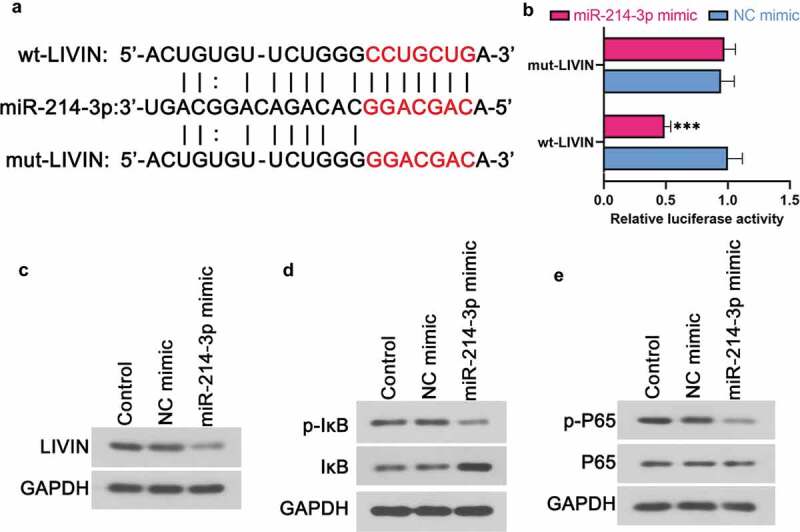
miR-214-3p affected LIVIN and its related signaling pathway. (a) Putative miR-214-3p binding and mutated sites in LIVIN. (b) Dual-luciferase reporter assay was performed to determine the luciferase activity of 293 T cells. (c-e) The protein levels of LIVIN (c), p-IκB (d), IκB (d), p-P65 (e) and P65 (e) in LoVo cells after the up-regulation of miR-214-3p. Data is shown as mean ± SD, *** p < 0.001.

Bioinformatic prediction indicated that LIVIN may bind to miR-214-3p (Figure 4(a)). As expected, the dual-luciferase reporter assay results confirmed that LIVIN could bind to miR-214-3p (Figure 3(b)). Western blot results indicated that the up-regulation of miR-214-3p could inhibit the protein expression of LIVIN in LoVo cells (Figure 4(c)). Moreover, miR-214-3p mimic could also reduce the phosphorylation of IκB (Figure 4(d)) and p65 (Figure 4(e)) in LoVo cells.

### DNAJC3-AS1/miR-214-3p axis affected the cell proliferation, invasion, and EMT of colon cancer cells

**Figure 5.**
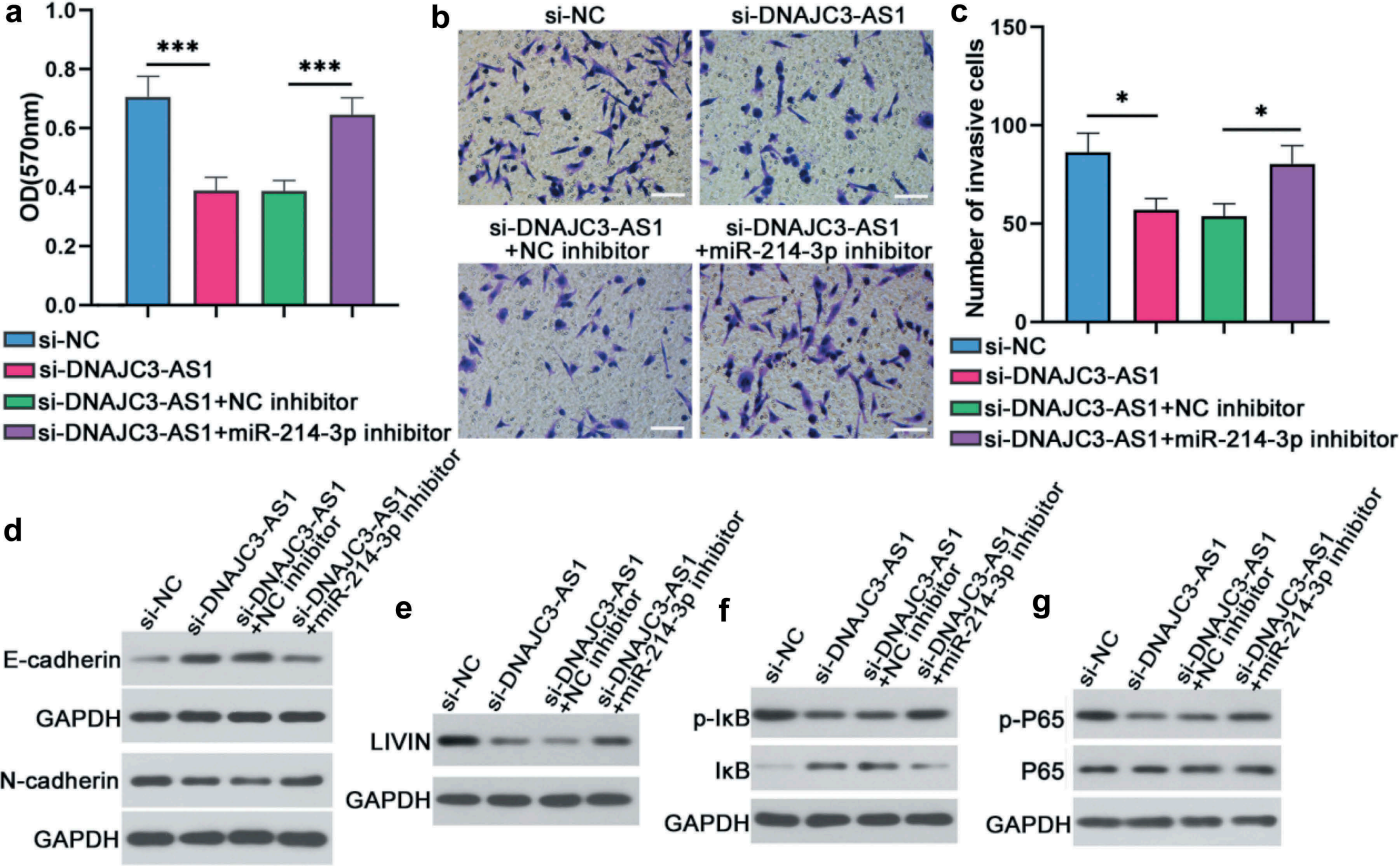
DNAJC3-AS1/miR-214-3p axis affected the cell proliferation, invasion, and EMT of colon cancer cells. (a) Cell proliferation of LoVo cells was analyzed by MTT assay. (b and c) Transwell assay was carried out to confirm cell invasive ability. Scale bar = 100 μm. (d-g) The protein levels of E-cadherin (d), N-cadherin (d), LIVIN (e), p-IκB (f), IκB (f), p-P65 (g) and P65 (g) in LoVo cells were detected by western blot. Data is shown as mean ± SD, * p < 0.05, *** p < 0.001.

MTT assay results revealed that down-regulation of DNAJC3-AS1 could inhibit the proliferation of LoVo cells, while down-regulation of miR-214-3p could increase the cell proliferation (Figure 5(a)). The invasion cell numbers of LoVo cells were reduced by si-DNAJC3-AS1, while this effect was abrogated by down-regulating miR-214-3p (Figure 5(b,c)). Besides, the protein expression of E-cadherin was increased by si-DNAJC3-AS1, while this increase could be reversed by a miR-214-3p inhibitor (Figure 5(d)). The expression of LIVIN was reduced by si-DNAJC3-AS1, while this effect was attenuated by a miR-214-3p inhibitor (Figure 5(e)). In addition, down-regulation of DNAJC3-AS1 could also reduce the phosphorylation of IκB and p65, while this effect was abrogated by a miR-214-3p inhibitor (figure 5(f,g)). These suggested that down-regulation of DNAJC3-AS1 could inhibit the cell proliferation, invasion and EMT of colon cancer cells through regulating miR-214-3p, and affect LIVIN expression, NF-κBp65 signaling through regulating miR-214-3p.

## Discussion

In cancer biology, the earliest evidence was the difference in gene expression between tumor tissues and normal tissues [25]. Previous studies indicated that lncRNA expression levels in tumors are significantly increased or decreased compared to normal tissues [25–27]. The basic hallmark capabilities of malignant transformation of cancer are: sustaining proliferation; evading growth suppressors; activating invasion and metastasis; inducing angiogenesis and resisting cell death [28]. It has been reported that some lncRNAs can affect cell proliferation [29], and participate in the complex interaction of cancer cells [30]. In addition, lncRNAs also play a role in tumor angiogenesis [30]. Therefore, lncRNAs can be used as novel biomarkers for diagnosis, prognosis, and treatment.

In this study, we found that lncRNA DNAJC3-AS1 expression was higher in colon tumor tissues than normal colon tissues. The overall survival of patients with the low expression levels of DNAJC3-AS1 was longer than that of patients with a strong expression level of DNAJC3-AS1. Moreover, the down-regulation of DNAJC3-AS1 inhibited the proliferation, migration, and invasion of colon cancer cells, and induced growth arrest *in vitro*. These findings indicated that DNAJC3-AS1 may play a direct role in colon cancer progression.

Inspired by the ‘competitive endogenous RNA’ regulatory network [14], we searched potential interactions with miRNAs. We used bioinformatics analysis and dual-luciferase reporter assays to verify the predicted binding capacity of miR-214-3p on DNAJC3-AS1. As expected, we found that miR-214-3p could complement base pairing with DNAJC3-AS1 and induce translational suppression of the RLuc-DNAJC3-AS1 reporter gene. qPCR results demonstrated that the expression of miR-124-3p was negatively correlated with the expression of DNAJC3-AS1 in colon cancer cells. Previous research has shown that miR-214 is a colon cancer inhibitor [31]. miR-214 inhibits colon cancer by down-regulating Wnt signaling in colon cancer [20]. Besides, miR-214 sensitizes colon cancer cells to 5-fluorouracil (5-FU) by targeting Hsp27 to overcome chemotherapy resistance [21]. Our results showed that knockdown of DNAJC3-AS1 inhibited the proliferation, invasion, and EMT of colon cancer cells, while these effects could be eliminated by miR-214-3p inhibitor. The data suggested that the DNAJC3-AS1/miR-214-3p axis may be related to the pathogenesis of colon cancer.

In order to study the mechanism of DNAJC3-AS1/miR-214-3p axis in colon cancer, we selected the predicted target gene LIVIN of miR-214-3p for further research. LIVIN is a member of the inhibitor of apoptosis proteins (IAPs) [32]. The expression of LIVIN is rarely found in normal tissues but is abundant in tumor tissues [32]. Overexpression of LIVIN protein has been reported in leukemia, hepatocellular carcinoma, and melanoma, colorectal cancer [33]. In addition, overexpression of LIVIN is associated with the high risk of bladder cancer recurrence [34]. Mountains studies have suggested that LIVIN could regulate the proliferation, migration, and invasion of cancer cells and act as an oncogene in various tumor cells [35–37]. More importantly, our team has shown that forced over-expression of LIVIN significantly promoted the proliferation, migration, invasion and EMT activities of colorectal cancer cells, on the contrary, siRNA-mediated knockdown of LIVIN had opposite effects [22]. In the present study, the down-regulation of DNAJC3-AS1 resulted in increased miR-214-3p level and reduced LIVIN expression. These results suggesting that DNAJC3-AS1 may affect the biological behavior of colorectal cancer cells through the regulation of LIVIN expression mediated by miR-214-3p.

The NF-kB pathway is considered to play pivotal roles in proliferation, apoptosis, invasion and other physiological processes of cancer cells [38,39]. The activation of the NF-kB pathway is tightly controlled by the IkB proteins, which inhibit the DNA binding activity and prevent the nuclear uptake of NF-kB complexes [40]. Mehrotra et al [41]. have proved that the IAPs contributes to tumor progression by the activation of the NF-kB pathway. Moreover, we have shown that over-expression of LIVIN promoted the nuclear transfer of NF-kB, and the NF-κB inhibitor BAY 11–7028 or P65 siRNA restores LIVIN-overexpression induced invasion and EMT of colorectal cells [22]. In this study, the down-regulation of DNAJC3-AS1 resulted in the reduction of LIVIN level, accompanied by the inactivation of NF-kB, as evidenced by the reduced p-IkB and p-P65 levels. In addition, these effects were obviously restored by the miR-214-3p inhibitor. These results indicate that the DNAJC3-AS1/miR-214-3p axis affects the activation of the NF-kB pathway via the regulation of LIVIN expression, which further controls the malignant phenotype of colorectal cancer cells.

## Conclusion

In summary, DNAJC3-AS1 was involved in cell proliferation, migration, invasion, and EMT of colon cancer cells. These effects were related to the activation of the NF-kB pathways that mediated by the miR-214-3p/LIVIN axis. These results enhanced our understanding of lncRNA functions, a better understanding of the pathogenesis of colon cancer, and promote the development of diagnosis and treatment of colon cancer.

## Highlights

- DNAJC3-AS1 affects the proliferation and metastatic ability of colon cancer cells.
- DNAJC3-AS1 functioned as a sponge of miR-214-3p in colon cancer cells.
- LIVIN was a target of miR-214-3p.
